# Supplementary material for: Electron affinity of liquid water
Source: Nat Commun. 2018 Jan 16;9:247. doi: 10.1038/s41467-017-02673-z (PMC5770385; doi:10.1038/s41467-017-02673-z)
Supplement: Supplementary file 1 — Supplementary Information [file 41467_2017_2673_MOESM1_ESM.pdf]

Supplementary Methods

Molecular Dynamics Simulations

Classical and path-integral (PI) molecular dynamics (MD) simulations were performed using the ab-initio MB-pol potential and a modified version of the DL_POLY code [1]. Bulk water was simulated in the constant volume-constant temperature (NVT) ensemble at 298 K, using a Nosé–Hoover chain thermostat [2] with the length of 4, and supercells containing 64 molecules. The equilibrium density (ρ) of the liquid was determined with constant pressure and temperature (NPT) simulations, corresponding to cell sizes of 12.41 Å^3 ($\rho = 1.001$ g cm^{-3}) and 12.42 Å^3 ($\rho = 0.999$ g cm^{-3}) for classical and PI molecular-dynamics simulations, respectively. Path-integral simulations were performed using 32 beads per atoms. The water surface was simulated with a slab containing 108-molecules, in a cell of dimensions $12.74 \times 12.74 \times 57.35$ Å^3. After equilibration, we carried out simulations for 0.5 and 1 ns for the bulk and the slab, respectively. In particular, we insured that the electric field across the water slab vanished within 0.0005 eV/Å. We tested size effects on the position of energy levels computed using the PBE functional, by performing MD simulations for 256-molecule bulk water and 216- and 384-molecule slabs. The lateral side of the bulk 256-molecule cell was 19.73 Å. The dimensions of the 216- and 324-molecule slabs were the same as that of the 108 molecule slab in the plane parallel to the surface, and 100 Å and 150 Å, respectively in the direction perpendicular to the surface.

Electronic Structure Calculations

Density-functional (DFT) and many-body-perturbation-theory calculations were performed on snapshots extracted from MD simulations. One hundred and twenty eight equally-spaced in time snapshots were extracted over 0.5- or 1-ns trajectories; when reducing the number of samples to 64 over the same trajectory length the average DFT orbital energies in bulk water changed by less than 0.03 eV, within the statistical error bars on the Kohn–Sham eigenvalues in our calculations (0.02–0.03 eV). The electronic properties of the liquid at the density-functional theory level were computed using the QUANTUM ESPRESSO code [3]. We set the kinetic energy cutoff to 85 Ry and used HSCV pseudopotentials [4–6]. G_0W_0 calculations were performed using the WEST code [7, 8]. Quasiparticle energies were determined using the same exchange-correlation potential as the one employed in the self-consistent-field DFT procedure. All our G_0W_0 calculations were carried out using $N_{\mathrm{PDEP}} = 1600$ eigenpotentials (with N_{PDEP} being the number of eigenpotentials used to represent the dielectric matrix; convergence with respect to the number of eigenpotentials is discussed below), and the results reported in the main text were extrapolated to the limit of infinite number of eigenpotentials using the function $a + b/N_{\mathrm{PDEP}}$.

Electronic densities of states (EDOS) were computed in the same way as in Supplementary Ref. 9, i.e. by summing Gaussian functions placed on each occupied and empty orbital energy level.

Size effects on the valence and conduction band edges

The single-particle orbital energies (ε_i) were computed using periodic boundary conditions and plane-wave basis sets; we carried out separate simulations for supercells representing bulk water and the surface of water (slab with the liquid in contact with vacuum) using the same interaction potential, MB-pol, and either path-integral or classical MD. The values ε_i obtained from a plane wave code (e.g. QUANTUM ESPRESSO) are defined with respect to an arbitrary zero of energy. To determine absolute energies with respect to vacuum, directly comparable to experiments, we followed the procedure [9, 10] originally proposed by van de Walle and Martin [11], and the absolute position of the energy levels in the slab ($\tilde{\varepsilon}_i^{\mathrm{slab}}$) and in the bulk ($\tilde{\varepsilon}_i^{\mathrm{bulk}}$) were obtained, respectively, as

$$\tilde{\varepsilon}_i^{\mathrm{slab}} = \varepsilon_i^{\mathrm{slab}} - V_{\mathrm{slab}}^{\mathrm{vacuum}} \tag{1}$$

and

$$\tilde{\varepsilon}_i^{\mathrm{bulk}} = \varepsilon_i^{\mathrm{bulk}} - \Delta V_{\mathrm{slab}} - V_{\mathrm{bulk}}. \tag{2}$$

In Eqs. (1) and (2), $\varepsilon_i^{\rm slab}$ and $\varepsilon_i^{\rm bulk}$ are energy levels referred to an arbitrary zero of energy; $\Delta V_{\rm slab} = V_{\rm slab}^{\rm vacuum} - V_{\rm slab}^{\rm water}$ is the difference between the plane-average electrostatic potential computed in the vacuum and water regions of the slab; $V_{\rm bulk}$ is the average electrostatic potential computed in bulk water. The values of all these quantities for quantum and classical water determined and utilized in this work are summarized in Supplementary Table 1.

We explored two ways of computing the valence and conduction band edges: (i) we equated the position of the valence band maximum (VBM) and conduction band minimum (CBM) to the highest occupied and lowest unoccupied energy levels obtained in our calculations; (ii) we extrapolated the peaks in the EDOS to zero energy. To do so, we computed the second derivative of the EDOS as a function of the single particle energy to find inflection points, followed by calculation of the tangent line, as shown in Supplementary Figure 1.

As pointed out by Ambrosio et al. [12], different definitions of the band edges have different rate of convergence with respect to the system size. To understand size effects, we first explored the convergence of EDOS at the DFT level with respect to the number of k points included in the electronic structure calculations. Supplementary Figure 2 shows that the EDOS of occupied states is insensitive to the number of k points, while the *shape* of the EDOS of the empty states substantially depends on the number of k points included in the calculation, as first noted in Supplementary Ref. 13. However the energies of the highest occupied (HOMO) and lowest unoccupied (LUMO) orbitals change by less than 0.01 eV when using 64 k-points compared to the Γ-point only calculation.

A comparison of the positions of HOMO and LUMO energies computed with cells of different size, as well as the extrapolated valence band edge are summarized in Supplementary Table 2. The Table shows that the HOMO energy exhibits slow convergence with respect to the system size [12]. The extrapolated position of the $1b_1$ band edge has instead a weaker system-size dependence.

On the other hand, extrapolation of the band edge for the conduction band minimum does not exhibit a satisfactory convergence trend with respect to the system size. This is shown in Supplementary Figure 3, which compares EDOS of unoccupied states of bulk water averaged along snapshots of the 64- and 256-molecule trajectories. Straightforward extrapolation of the conduction band minimum using the first, LUMO peak of the EDOS yields −2.54 eV for the 64-molecule system and −2.60 eV for the 256-molecule system, respectively. These values are substantially lower in energy (by $\sim$ 0.3 eV) than those obtained using the energies of the LUMO states, −2.22 and −2.29 eV respectively (Supplementary Table 2). Since the converged dependence of the unoccupied EDOS as a function of energy is close to linear, extrapolations should be performed over a larger energy range, and not just by considering the first peak. We verified that when considering a larger energy range between −2.4 and −2.8 eV (see Supplementary Figure 2), the extrapolation of the EDOS computed at Γ and with 64 k-points yields a value for the band edge which is within 0.05 eV and 0.005 eV of that obtained considering the LUMO energy, respectively.

The HOMO energy computed for slabs behaves similarly to that computed for bulk water, as a function of the supercell size (see Supplementary Table 2). However we note that the fluctuations of the energy of the valence and conduction band edges as a function of the simulation time are much larger for slabs (0.4–0.6 eV) than in bulk samples (0.1–0.4 eV), leading to a larger error estimate of the valence and conduction band edges near the water surface, compared to the bulk. The LUMO energy of the slab converges slower with respect to the system size than that of the bulk, and it appears to be converged for the 216-water-molecule sample, within statistical error bars of 0.1 eV. Since performing hybrid DFT and G_0W_0 calculations for 216-molecule slabs was prohibitively expensive, we computed band edges for the 108-molecule slabs at PBE, PBE0, RSH, and sc-hybrid levels of theory, and corrected the position of the CBM by the difference between the LUMO of the 108- and 216-molecule slabs (−0.22 eV), computed with the PBE functional. No size corrections were necessary for the extrapolated $1b_1$ edge position for the 108-molecule supercell.

Based on the results reported in this section, we used a linearly extrapolated edge of the $1b_1$ peak as a measure of the valence band maximum of both the bulk liquid and the surface. We used instead the LUMO energy as a definition of the conduction band minimum, corrected by −0.22 eV in the case

of the water slab to take into account size effects, as discussed above.

G_0W_0 calculations

We performed G_0W_0 calculations starting with converged PBE and hybrid functional wavefunctions for classical and quantum bulk water. G_0W_0/PBE calculations were carried out for 128 snapshots along each trajectory, while the G_0W_0 calculations starting with hybrid functionals were performed for 4 representative snapshots selected from the quantum and classical trajectories. Supplementary Tables 3 and 4 show that the statistical error for LUMO estimated over a set of 4 snapshots is up to 0.1 eV, while the statistical error for the energy differences ΔG_0W_0 is smaller, up to 0.03 eV. Therefore we computed the G_0W_0/hybrid conduction band edge by correcting the DFT LUMO energy with ΔG_0W_0 values from Supplementary Tables 3 and 4. The statistical error bars on the resulting CBM were estimated as the sum of the error of the DFT energy and the error of the correction ΔG_0W_0. The valence band edge was always determined using the extrapolated $1b_1$ peak of the G_0W_0/hybrid EDOS, without applying any corrections.

We also investigated the convergence of the G_0W_0 energy of the LUMO orbital with respect to the number of eigenpotentials, N_{PDEP}. We computed the LUMO energy of a single quantum water snapshot using G_0W_0/PBE as a function of the number of eigenpotentials (Supplementary Table 5). We then fit the resulting LUMO energies to the function $a + b/N_{\mathrm{PDEP}}$ where a is the LUMO energy at infinite number of eigenpotentials. We find that the extrapolation procedure lowers the LUMO energy by 0.32 eV compared to calculations with 1600 eigenpotentials performed in this work. This correction of -0.32 eV determined at G_0W_0/PBE level of theory was applied to all G_0W_0 LUMO energies computed in this work.

G_0W_0 calculations for the water/vacuum interface were prohibitive, from a computational standpoint, if conduced at the same statistical level as for the bulk water. Hence, we obtained G_0W_0 energies for the slab in the same way as we did for the G_0W_0/hybrid results—DFT energies computed for the slab, plus the ΔG_0W_0 correction from Supplementary Table 3 and 4, extrapolated to the infinite number of eigenpotentials. The analysis provided above showed that this scheme adds a modest additional statistical error of ~ 0.03 eV. To check that this protocol still applies to water in contact with vacuum, we performed G_0W_0/PBE calculations for one snapshot of the quantum water/vacuum interface at several numbers of eigenpotentials, and then extrapolated the LUMO energy to the infinite N_{PDEP}. The results, shown in Supplementary Table 6, indicate that $\varepsilon_{\mathrm{LUMO}}$ computed as a correction to the DFT energy differs from the extrapolated G_0W_0 value by 0.01 eV, thus justifying our computational procedure.

Supplementary Note

This Section summarizes the values of the electronic properties of classical and quantum bulk water (Supplementary Tables 7 and 8), as well as water/vacuum interface (Supplementary Tables 9 and 10).

Nuclear quantum effects (NQE) on water structure have been discussed in Supplementary Ref. 14. Here we report on NQE influence on the electronic structure. Supplementary Figure 4 compares the experimental photoelectron spectrum to the density of states, computed following the protocol and the best level of theory (G_0W_0 quasiparticle energies obtained using RSH wavefunctions) presented in Supplementary Refs. 9 and 15. The Supplementary Figure 4 shows that NQE have a softening effect on the shape of the spectrum. The width of the bands increases and their magnitude decreases, consistent with the recent study of Supplementary Ref. 16. Overall, the spectrum computed over the PIMD trajectory appears to be closer to experimental results, although photoionization cross-sections would need to be taken into account for a full detailed comparison [9].

Supplementary Figures 5 and 6 compare electronic properties (effective molecular polarizabilities, dipole moments, distribution of Wannier centers) of water molecules in bulk water (classical and quantum) and at the interface of quantum water with vacuum.

Supplementary Table 1: Electrostatic potential in the water ($V_{\text{slab}}^{\text{water}}$) and vacuum ($V_{\text{slab}}^{\text{vacuum}}$) portions of the slab simulated with (Quantum water) and without (Classical water) nuclear quantum effects, computed with different density functionals (PBE [17], PBE0 [18], RSH [19], and sc-hybrid [20]). The sum of $\Delta V_{\text{slab}} = V_{\text{slab}}^{\text{vacuum}} - V_{\text{slab}}^{\text{water}}$ and V_{bulk} (0.12 eV) used in Eq. (2) is also reported. G_0W_0/DFT eigenvalues were aligned with vacuum using respective DFT values. All values are in eV.

	Quantum water			Classical water		
Density functional	$V_{\text{slab}}^{\text{vacuum}}$	$V_{\text{slab}}^{\text{water}}$	$\Delta V_{\text{slab}} + V_{\text{bulk}}$	$V_{\text{slab}}^{\text{vacuum}}$	$V_{\text{slab}}^{\text{water}}$	$\Delta V_{\text{slab}} + V_{\text{bulk}}$
PBE	1.41	-2.45	3.98	1.42	-2.43	3.97
PBE0	1.39	-2.40	3.90	1.39	-2.38	3.90
RSH	1.37	-2.37	3.86	1.38	-2.36	3.86
sc-hybrid	1.35	-2.33	3.80	1.36	-2.33	3.82

Supplementary Table 2: Average energies of the highest occupied (HOMO) and lowest unoccupied (LUMO) orbitals computed with the PBE functional along quantum MB-pol trajectories of bulk water and slab with different number of molecules in the periodic cell. The position of the edge was computed by extrapolation of the $1b_1$ peak of the electronic density of states (EDOS), as discussed in Supplementary Methods. The error bars were computed as three standard deviations of the mean across the set of snapshots picked from each trajectory. The error bars on the $1b_1$ peak edges could not be determined as they are extrapolated from the average EDOS, not from EDOS of individual snapshots. All values are in eV.

System	Size	Edge ($1b_1$), eV	$\varepsilon_{\mathrm{HOMO}}$, eV	$\varepsilon_{\mathrm{LUMO}}$, eV
Bulk	64	-6.13	-6.09 ± 0.05	-2.22 ± 0.02
	256	-6.10	-5.81 ± 0.05	-2.29 ± 0.01
Slab	108	-6.17	-5.98 ± 0.08	-2.41 ± 0.05
	216	-6.05	-5.81 ± 0.11	-2.63 ± 0.07
	324	-5.93	-5.64 ± 0.11	-2.78 ± 0.10

Supplementary Table 3: Density-functional theory (DFT) and G_0W_0/DFT lowest unoccupied molecular orbital (LUMO) energies averaged for select snapshots extracted from the classical MB-pol trajectory for bulk water. ΔG_0W_0 refers to differences between the G_0W_0 quasiparticle energies and the respective DFT eigenvalue, and represents a correction provided by the G_0W_0 method to the LUMO energy. "Average (4 samples)" denotes the average computed using 4 snapshots taken from each trajectory, while "Average (trajectory)" refers to the average computed along the entire trajectory (128 snapshots). The latter is available for all DFT methods and for G_0W_0/PBE, but is not available for G_0W_0/hybrid calculations. All errors are computed as three standard deviations of the mean on the respective quantity. The energies (in eV) are given on an absolute scale, referred to vacuum using Eq. (2) and values from Supplementary Table 1. PBE, PBE0, RSH, and sc-hybrid functionals are defined in Supplementary Refs. 17, 18, 19, and 20, respectively.

Method	Average (4 samples)	Average (trajectory)
PBE	-1.99 ± 0.07	-2.00 ± 0.01
G_0W_0/PBE	-0.66 ± 0.05	-0.67 ± 0.01
ΔG_0W_0/PBE	1.33 ± 0.03	1.329 ± 0.003
PBE0	-1.17 ± 0.07	-1.18 ± 0.01
G_0W_0/PBE0	-0.13 ± 0.05	—
ΔG_0W_0/PBE0	1.04 ± 0.02	—
RSH	-0.15 ± 0.07	-0.16 ± 0.01
G_0W_0/RSH	0.23 ± 0.06	—
ΔG_0W_0/RSH	0.38 ± 0.02	—
sc-hybrid	-0.13 ± 0.07	-0.14 ± 0.01
G_0W_0/sc-hybrid	0.32 ± 0.06	—
ΔG_0W_0/sc-hybrid	0.45 ± 0.02	—

Supplementary Table 4: Same quantities as reported in Supplementary Table 3 but for the quantum MB-pol trajectory for bulk water. ΔG_0W_0 refers to differences between the G_0W_0 quasiparticle energies and the respective DFT eigenvalue, and represents a correction provided by the G_0W_0 method. "Average (4 samples)" denotes the average computed using 4 snapshots taken from each trajectory, while "Average (trajectory)" refers to the average computed along the entire trajectory (128 snapshots). The latter is available for all DFT methods and for G_0W_0/PBE, but not for G_0W_0/hybrid calculations. All errors were computed as three standard deviations of the mean on the respective quantity. The energies (in eV) are given on an absolute scale, referred to vacuum using Eq. (2) and values from Supplementary Table 1. PBE, PBE0, RSH, and sc-hybrid functionals are defined in Supplementary Refs. 17, 18, 19, and 20, respectively.

Method	Average (4 samples)	Average (trajectory)
PBE	-2.19 ± 0.10	-2.21 ± 0.02
G_0W_0/PBE	-0.83 ± 0.07	-0.85 ± 0.01
ΔG_0W_0/PBE	1.36 ± 0.03	1.362 ± 0.006
PBE0	-1.35 ± 0.09	-1.38 ± 0.02
G_0W_0/PBE0	-0.30 ± 0.07	—
ΔG_0W_0/PBE0	1.05 ± 0.03	—
RSH	-0.33 ± 0.09	-0.35 ± 0.02
G_0W_0/RSH	0.06 ± 0.07	—
ΔG_0W_0/RSH	0.39 ± 0.02	—
sc-hybrid	-0.29 ± 0.08	-0.31 ± 0.02
G_0W_0/sc-hybrid	0.17 ± 0.07	—
ΔG_0W_0/sc-hybrid	0.46 ± 0.02	—

Supplementary Table 5: Convergence of the G_0W_0/PBE quasiparticle energy of the lowest unoccupied state $\varepsilon_{\mathrm{LUMO}}$ as a function of the number of eigenpotentials N_{PDEP} for a snapshot of quantum bulk water. The symbol ∞ denotes the value obtained by linear extrapolation with respect to $1/N_{\mathrm{PDEP}}$, and represents a fully converged G_0W_0 result. All values are in eV.

N_{PDEP}	$\varepsilon_{\mathrm{LUMO}}$, eV
1600	−0.83
2048	−0.91
2560	−0.95
3072	−0.98
3584	−1.01
	⋮
∞	−1.15

Supplementary Table 6: Convergence of the G_0W_0/PBE quasiparticle energy of the lowest unoccupied state $\varepsilon_{\mathrm{LUMO}}$ as a function of the number of eigenpotentials N_{PDEP} for a snapshot of quantum water surface. The symbol ∞ denotes the value obtained by linear extrapolation with respect to $1/N_{\mathrm{PDEP}}$, and represents a fully converged G_0W_0/PBE result. "$\mathrm{PBE} + \Delta G_0W_0$" represents the estimate computed as a DFT energy plus a G_0W_0 correction from Supplementary Table 4, additionally extrapolated to the infinite number of eigenpotentials by adding -0.32 eV, as determined in Supplementary Table 5. All values are in eV.

N_{PDEP}	$\varepsilon_{\mathrm{LUMO}}$, eV
1600	-1.05
2048	-1.16
2560	-1.24
3072	-1.33
3584	-1.36
⋮	
∞	-1.61
$\mathrm{PBE} + \Delta G_0W_0$	-1.60

Supplementary Table 7: Positions of peaks in the electronic densities of states (EDOS) computed along the classical MB-pol trajectory of bulk liquid water using a series of density functionals (PBE [17], PBE0 [18], RSH [19], and sc-hybrid [20]) and many-body perturbation theory (G_0W_0). The mean absolute error (MAE) of peak positions reflects the average deviation of computed peaks from the experimental ones. The valence band maximum (VBM) was determined by the linear extrapolation of the $1b_1$ peak of water to zero. The conduction band minimum (CBM) is the LUMO energy, and in the case of G_0W_0 calculations were extrapolated to infinite number of eigenpotentials. The energies were aligned with respect to vacuum using Eq. (2) and the values from Supplementary Table 1, for all DFT and G_0W_0 orbital energies obtained in plane-wave calculations. The energies are in eV.

	Peak positions, eV						
Method	$2a_1$	$1b_2$	$3a_1$	$1b_1$	MAE	VBM	CBM
PBE	−25.05	−13.03	−9.17	−7.32	4.70	−6.35	-2.00 ± 0.01
PBE0	−28.24	−14.88	−10.98	−9.07	2.56	−8.09	-1.18 ± 0.01
RSH	−30.76	−16.77	−12.80	−10.89	0.55	−9.89	-0.16 ± 0.01
sc-hybrid	−32.32	−17.24	−13.31	−11.35	0.52	−10.35	-0.14 ± 0.01
G_0W_0/PBE	−29.38	−16.40	−12.43	−10.21	1.24	−9.20	-0.99 ± 0.01[c]
G_0W_0/PBE0	−29.92	−17.29	−13.41	−11.07	0.43	−10.00[a]	-0.46 ± 0.04[b,c]
G_0W_0/RSH	−31.48	−17.86	−13.95	−11.61	0.38	−10.53[a]	-0.10 ± 0.03[b,c]
G_0W_0/sc-hybrid	−31.76	−17.92	−14.03	−11.68	0.50	−10.60[a]	-0.01 ± 0.03[b,c]
Experiment	−30.90[d]	−17.409[e]	−13.78[f]	−11.31[e]	—	−9.9[d], −10.06[g]	—

[a]Obtained as $1b_1$ peak edge of EDOS averaged over 4 snapshots.
[b]DFT LUMO energy corrected by a ΔG_0W_0 value from the Supplementary Table 3.
[c]Value lowered by 0.32 eV to extrapolate to infinite number of eigenpotentials (see Supplementary Table 5).
[d]Supplementary Ref. 21.
[e]Supplementary Ref. 22.
[f]Average of $3a_1$H and $3a_1$L values in Supplementary Ref. 22.
[g]Supplementary Refs. 23 and 24.

Supplementary Table 8: Positions of peaks in the electronic densities of states (EDOS) computed along the quantum MB-pol trajectory of bulk liquid water using a series of density functionals (PBE [17], PBE0 [18], RSH [19], and sc-hybrid [20]) and many-body perturbation theory (G_0W_0). The mean absolute error (MAE) of peak positions reflects the average deviation of computed peaks from the experimental ones. The valence band maximum (VBM) was determined by the linear extrapolation of the $1b_1$ peak of water to zero. The conduction band minimum (CBM) is the LUMO energy, and in the case of G_0W_0 calculations extrapolated to infinite number of eigenpotentials. The energies were aligned with respect to vacuum using Eq. (2) and the values from Supplementary Table 1, for all DFT and G_0W_0 orbital energies obtained in plane-wave calculations. The energies are in eV.

	Peak positions, eV						
Method	$2a_1$	$1b_2$	$3a_1$	$1b_1$	MAE	VBM	CBM
PBE	−25.00	−12.85	−9.23	−7.26	4.76	−6.13	-2.22 ± 0.02
PBE0	−28.19	−14.69	−11.03	−8.99	2.62	−7.85	-1.38 ± 0.02
RSH	−30.71	−16.57	−12.85	−10.80	0.62	−9.64	-0.35 ± 0.02
sc-hybrid	−32.24	−17.02	−13.33	−11.24	0.56	−10.08	-0.31 ± 0.02
G_0W_0/PBE	−29.12	−16.25	−12.27	−10.09	1.42	−8.88	-1.17 ± 0.01^{c}
G_0W_0/PBE0	−29.83	−16.82	−13.15	−10.98	0.66	-9.70^{a}	$-0.65 \pm 0.04^{b,c}$
G_0W_0/RSH	−31.32	−17.37	−13.71	−11.53	0.18	-10.24^{a}	$-0.29 \pm 0.05^{b,c}$
G_0W_0/sc-hybrid	−31.57	−17.42	−13.77	−11.57	0.24	-10.28^{a}	$-0.17 \pm 0.04^{b,c}$
Experiment	-30.90^{d}	-17.409^{e}	-13.78^{f}	-11.31^{e}	—	-9.9^{d}, -10.06^{g}	—

[a] Obtained as $1b_1$ peak edge of EDOS averaged over 4 snapshots.
[b] DFT LUMO energy corrected by a ΔG_0W_0 value from the Supplementary Table 4.
[c] Value lowered by 0.32 eV to extrapolate to infinite number of eigenpotentials (see Supplementary Table 5).
[d] Supplementary Ref. 21.
[e] Supplementary Ref. 22.
[f] Average of $3a_1$H and $3a_1$L values in Supplementary Ref. 22.
[g] Supplementary Refs. 23 and 24.

Supplementary Table 9: Positions of peaks in the electronic densities of states (EDOS) computed along the classical MB-pol trajectory of water/vacuum interface using a series of density functionals (PBE [17], PBE0 [18], RSH [19], and sc-hybrid [20]) and many-body perturbation theory (G_0W_0). The mean absolute error (MAE) of peak positions reflects the average deviation of computed peaks from the experimental ones. The valence band maximum (VBM) was determined by the linear extrapolation of the $1b_1$ peak of water to zero. The conduction band minimum (CBM) is the LUMO energy. No EDOS peak positions were reported for G_0W_0 results because ΔG_0W_0 were determined only for VBM and CBM energy levels. The G_0W_0 LUMO energies were additionally corrected for the size of the slab (-0.22 eV) and extrapolated to the infinite number of eigenpotentials (-0.32 eV). The accuracy of this approximation is discussed in the preceding sections. The energies were aligned with respect to vacuum using Eq. (1) and the $V_{\mathrm{slab}}^{\mathrm{vacuum}}$ values from Supplementary Table 1, for all DFT and G_0W_0 orbital energies obtained in plane-wave calculations. All energies are in eV.

	Peak positions, eV						
Method	$2a_1$	$1b_2$	$3a_1$	$1b_1$	MAE	VBM	CBM
PBE	−25.25	−13.19	−9.42	−7.45	4.52	−6.44	-2.42^{a}
PBE0	−28.44	−15.03	−11.23	−9.20	2.37	−8.17	-1.64^{a}
RSH	−30.96	−16.92	−13.06	−11.02	0.39	−9.97	-0.78^{a}
sc-hybrid	−32.51	−17.39	−13.57	−11.48	0.50	−10.43	-0.76^{a}
G_0W_0/PBE						-9.29^{b}	$-1.42^{a,c,d}$
G_0W_0/PBE0						-10.08^{b}	$-0.92^{a,c,d}$
G_0W_0/RSH						-10.61^{b}	$-0.72^{a,c,d}$
G_0W_0/sc-hybrid						-10.68^{b}	$-0.63^{a,c,d}$
Experiment	-30.90^{e}	-17.409^{f}	-13.78^{g}	-11.31^{f}	—	-9.9^{e}, -10.06^{h}	—

[a] Value lowered by 0.22 eV to correct for the slab size (Supplementary Table 2).
[b] DFT valence band edge corrected using the difference of DFT and G_0W_0 VBM values from Supplementary Table 7.
[c] DFT conduction band minimum corrected using ΔG_0W_0 derived for LUMO in Supplementary Table 3.
[d] Value lowered by 0.32 eV to extrapolate to the infinite number of eigenpotentials (Supplementary Table 5).
[e] Supplementary Ref. 21.
[f] Supplementary Ref. 22.
[g] Average of $3a_1$H and $3a_1$L values in Supplementary Ref. 22.
[h] Supplementary Refs. 23 and 24.

Supplementary Table 10: Positions of peaks in the electronic densities of states (EDOS) computed along the quantum MB-pol trajectory of water/vacuum interface using a series of density functionals (PBE [17], PBE0 [18], RSH [19], and sc-hybrid [20]) and many-body perturbation theory (G_0W_0). The mean absolute error (MAE) of peak positions reflects the average deviation of computed peaks from the experimental ones. The valence band maximum (VBM) was determined by the linear extrapolation of the $1b_1$ peak of water to zero. The conduction band minimum (CBM) is the LUMO energy. No EDOS peak positions were reported for G_0W_0 results because ΔG_0W_0 were determined only for VBM and CBM energy levels. The G_0W_0 LUMO energies were additionally corrected for the size of the slab (−0.22 eV) and extrapolated to the infinite number of eigenpotentials (−0.32 eV). The accuracy of this approximation is discussed in the preceding sections. The energies were aligned with respect to vacuum using Eq. (1) and the $V_{\text{slab}}^{\text{vacuum}}$ values from Supplementary Table 1, for all DFT and G_0W_0 orbital energies obtained in plane-wave calculations. All energies are in eV.

	Peak positions, eV						
Method	$2a_1$	$1b_2$	$3a_1$	$1b_1$	MAE	VBM	CBM
PBE	−25.15	−13.09	−9.47	−7.42	4.57	−6.17	-2.63^{a}
PBE0	−28.33	−14.93	−11.26	−9.16	2.43	−7.89	-1.84^{a}
RSH	−30.84	−16.82	−13.06	−10.97	0.43	−9.68	-0.95^{a}
sc-hybrid	−32.38	−17.28	−13.57	−11.41	0.48	−10.13	-0.92^{a}
G_0W_0/PBE						-8.92^{b}	$-1.59^{a,c,d}$
G_0W_0/PBE0						-9.74^{b}	$-1.11^{a,c,d}$
G_0W_0/RSH						-10.28^{b}	$-0.88^{a,c,d}$
G_0W_0/sc-hybrid						-10.34^{b}	$-0.79^{a,c,d}$
Experiment	-30.90^{e}	-17.409^{f}	-13.78^{g}	-11.31^{f}	—	-9.9^{e}, -10.06^{h}	—

[a] Value lowered by 0.22 eV to correct for the slab size (Supplementary Table 2).
[b] DFT valence band edge corrected using the difference of DFT and G_0W_0 VBM values from Supplementary Table 8.
[c] DFT conduction band minimum corrected using ΔG_0W_0 derived for LUMO in Supplementary Table 4.
[d] Value lowered by 0.32 eV to extrapolate to the infinite number of eigenpotentials (Supplementary Table 5).
[e] Supplementary Ref. 21.
[f] Supplementary Ref. 22.
[g] Average of $3a_1$H and $3a_1$L values in Supplementary Ref. 22.
[h] Supplementary Refs. 23 and 24.

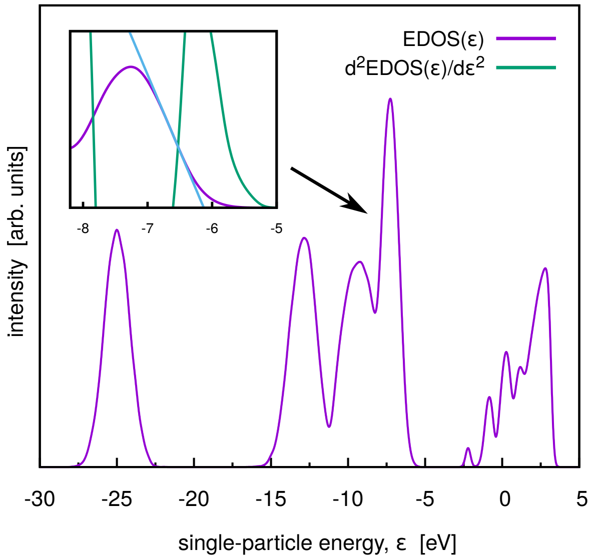

Supplementary Figure 1: Graphical representation of our calculation of the valence band edge by extrapolating the $1b_1$ peak of the electronic density of states (EDOS) as a function of the single-particle energy (ε). EDOS(ε) was obtained using the PBE functional along the quantum MB-pol trajectory of bulk water in the 64-molecule supercell.

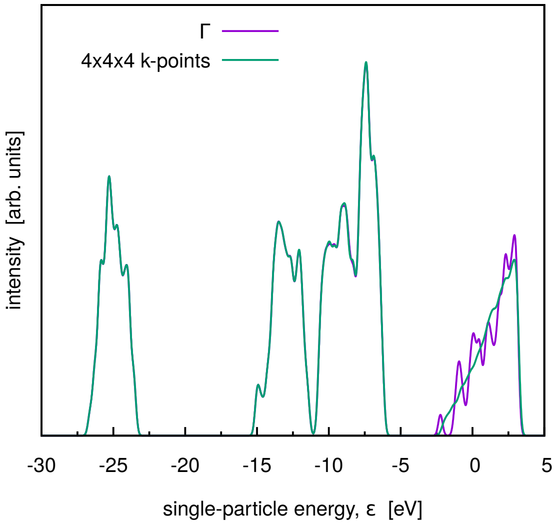

Supplementary Figure 2: Convergence of the electronic density of states (EDOS) with respect to the number of k points for a single molecular snapshot taken from the quantum MB-pol trajectory of bulk water. Calculations were done using the PBE functional.

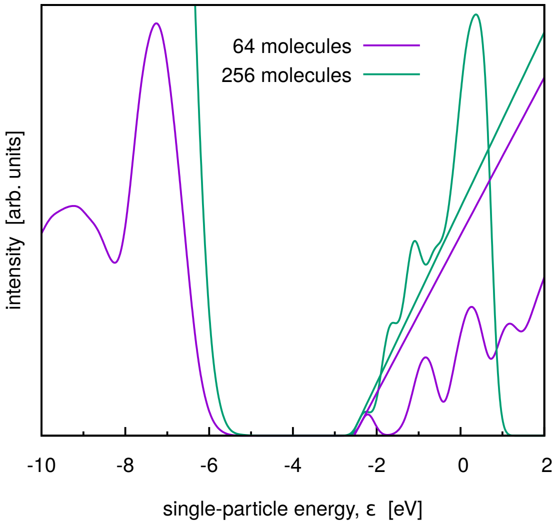

Supplementary Figure 3: Convergence of the electronic density of states (EDOS) with respect to the size of the unit cell. EDOS was computed using the PBE functional along the quantum MB-pol trajectory of bulk water represented by 64- and 256-molecule supercells.

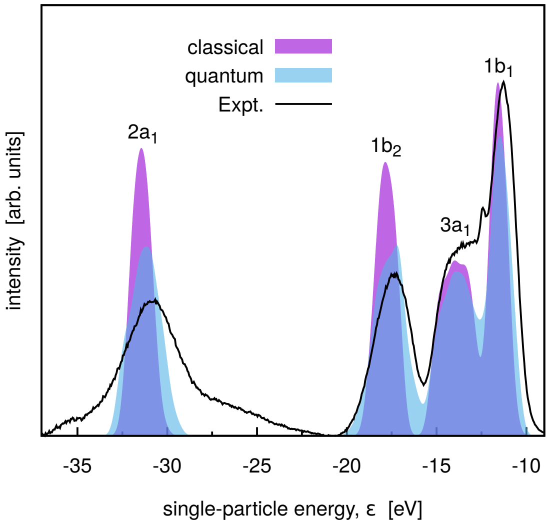

Supplementary Figure 4: Density of electronic states (EDOS) as a function of energy (ε) obtained using G_0W_0/RSH calculations for snapshots extracted from MB-pol trajectories of water with and without nuclear quantum effects. The experimental photoelectron spectrum is from Supplementary Ref. 21.

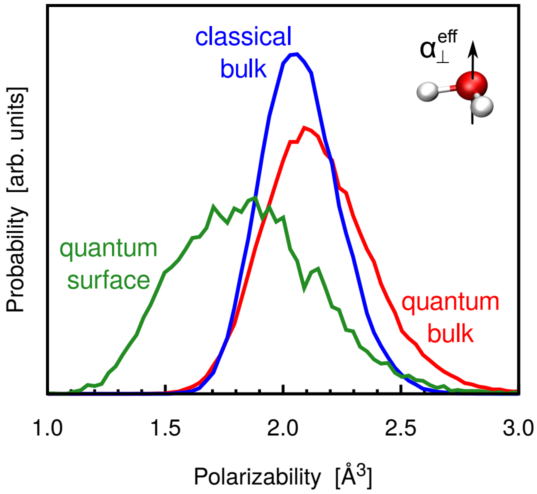

Supplementary Figure 5: Effective molecular polarizabilities $\alpha_{\perp}^{\mathrm{eff}}$ projected on the axis perpendicular to the molecular plane. The polarizabilities were calculated from first principles using the PBE functional, following Supplementary Ref. 25. Bulk water and slab were simulated with the MB-pol potential, and nuclear quantum effects were included.

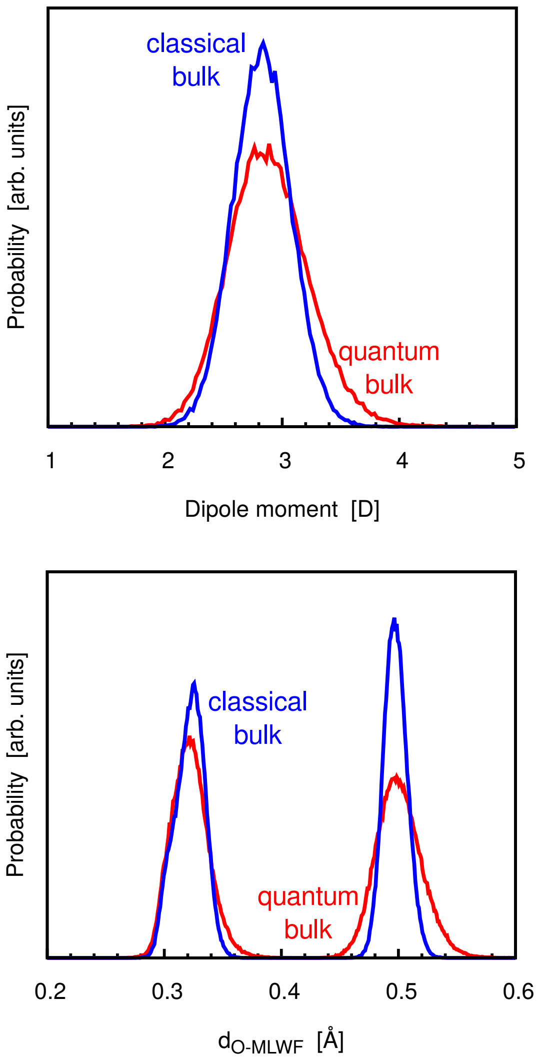

Supplementary Figure 6: Electronic properties of classical and quantum bulk water—distribution of dipole moments (top panel) and of distances from oxygen atoms to maximally localized Wannier centers [26, 27] (bottom panel). The peak at larger $d_{\mathrm{O-MLWF}}$ values corresponds to bond pairs and the one at shorter distances to lone pairs.

Supplementary References

[1] DL_POLY code (version 2.0): https://ccpforge.cse.rl.ac.uk/gf/project/dl_poly_classic/ (retrieved November 29, 2017).

[2] G. J. Martyna, M. L. Klein, and M. Tuckerman, "Nosé–Hoover chains: The canonical ensemble via continuous dynamics", *J. Chem. Phys.* **97**, 2635 (1992).

[3] P. Giannozzi, S. Baroni, N. Bonini, M. Calandra, R. Car, C. Cavazzoni, D. Ceresoli, G. L. Chiarotti, M. Cococcioni, I. Dabo, A. Dal Corso, S. de Gironcoli, S. Fabris, G. Fratesi, R. Gebauer, U. Gerstmann, C. Gougoussis, A. Kokalj, M. Lazzeri, L. Martin-Samos, N. Marzari, F. Mauri, R. Mazzarello, S. Paolini, A. Pasquarello, L. Paulatto, C. Sbraccia, S. Scandolo, G. Sclauzero, A. P. Seitsonen, A. Smogunov, P. Umari, and R. M. Wentzcovitch, "Quantum Espresso: a modular and open-source software project for quantum simulations of materials", *J. Phys. Condens. Matter* **21**, 395502 (2009).

[4] Pseudopotential table: http://fpmd.ucdavis.edu/potentials/index.htm (retrieved November 29, 2017).

[5] D. R. Hamann, M. Schlüter, and C. Chiang, "Norm-conserving pseudopotentials", *Phys. Rev. Lett.* **43**, 1494 (1979).

[6] D. Vanderbilt, "Optimally smooth norm-conserving pseudopotentials", *Phys. Rev. B* **32**, 8412 (1985).

[7] M. Govoni and G. Galli, "Large scale GW calculations", *J. Chem. Theory Comput.* **11**, 2680 (2015).

[8] WEST code (version 1.0.1): http://west-code.org/ (retrieved November 29, 2017).

[9] A. P. Gaiduk, M. Govoni, R. Seidel, J. H. Skone, B. Winter, and G. Galli, "Photoelectron spectra of aqueous solutions from first principles", *J. Am. Chem. Soc.* **138**, 6912 (2016).

[10] T. A. Pham, C. Zhang, E. Schwegler, and G. Galli, "Probing the electronic structure of liquid water with many-body perturbation theory", *Phys. Rev. B* **89**, 060202(R) (2014).

[11] C. G. Van de Walle and R. M. Martin, "Theoretical study of band offsets at semiconductor interfaces", *Phys. Rev. B* **35**, 8154 (1987).

[12] F. Ambrosio, G. Miceli, and A. Pasquarello, "Redox levels in aqueous solution: Effect of van der Waals interactions and hybrid functionals", *J. Chem. Phys.* **143**, 244508 (2015).

[13] D. Prendergast, J. C. Grossman, and G. Galli, "The electronic structure of liquid water within density-functional theory", *J. Chem. Phys.* **123**, 014501 (2005).

[14] G. R. Medders, V. Babin, and F. Paesani, "Development of a "first-principles" water potential with flexible monomers. III. Liquid phase properties", *J. Chem. Theory Comput.* **10**, 2906 (2014).

[15] T. A. Pham, M. Govoni, R. Seidel, S. E. Bradforth, E. Schwegler, and G. Galli, "Electronic structure of aqueous solutions: Bridging the gap between theory and experiments", *Sci. Adv.* **3**, e1603210 (2017).

[16] W. Chen, F. Ambrosio, G. Miceli, and A. Pasquarello, "Ab initio electronic structure of liquid water", *Phys. Rev. Lett.* **117**, 186401 (2016).

[17] J. P. Perdew, K. Burke, and M. Ernzerhof, "Generalized gradient approximation made simple", *Phys. Rev. Lett.* **77**, 3865 (1996).

[18] C. Adamo and V. Barone, "Toward reliable density functional methods without adjustable parameters: The PBE0 model", *J. Chem. Phys.* **110**, 6158 (1999).

[19] J. H. Skone, M. Govoni, and G. Galli, "Nonempirical range-separated hybrid functionals for solids and molecules", *Phys. Rev. B* **93**, 235106 (2016).

[20] J. H. Skone, M. Govoni, and G. Galli, "Self-consistent hybrid functional for condensed systems", *Phys. Rev. B* **89**, 195112 (2014).

[21] B. Winter, R. Weber, W. Widdra, M. Dittmar, M. Faubel, and I. V. Hertel, "Full valence band photoemission from liquid water using EUV synchrotron radiation", *J. Phys. Chem. A* **108**, 2625 (2004).

[22] N. Kurahashi, S. Karashima, Y. Tang, T. Horio, B. Abulimiti, Y.-I. Suzuki, Y. Ogi, M. Oura, and T. Suzuki, "Photoelectron spectroscopy of aqueous solutions: Streaming potentials of NaX (X = Cl, Br, and I) solutions and electron binding energies of liquid water and X^-", *J. Chem. Phys.* **140**, 174506 (2014).

[23] P. Delahay and K. Von Burg, "Photoelectron emission spectroscopy of liquid water", *Chem. Phys. Lett.* **83**, 250 (1981).

[24] P. Delahay, "Photoelectron emission spectroscopy of aqueous solutions", *Acc. Chem. Res.* **15**, 40 (1982).

[25] Q. Wan, L. Spanu, G. A. Galli, and F. Gygi, "Raman Spectra of Liquid Water from *Ab Initio* Molecular Dynamics: Vibrational Signatures of Charge Fluctuations in the Hydrogen Bond Network", *J. Chem. Theory Comput.* **9**, 4124 (2013).

[26] F. Gygi, J.-L. Fattebert, and E. Schwegler, "Computation of Maximally Localized Wannier Functions using a simultaneous diagonalization algorithm", *Comput. Phys. Commun.* **155**, 1 (2003).

[27] N. Marzari, A. A. Mostofi, J. R. Yates, I. Souza, and D. Vanderbilt, "Maximally localized Wannier functions: Theory and applications", *Rev. Mod. Phys.* **84**, 1419 (2012).
